# Closed Form Solutions for Unsteady Free Convection Flow of a Second Grade Fluid over an Oscillating Vertical Plate

**DOI:** 10.1371/journal.pone.0085099

**Published:** 2014-02-14

**Authors:** Farhad Ali, Ilyas Khan, Sharidan Shafie

**Affiliations:** 1 Department of Mathematical Sciences, Faculty of Science, Universiti Teknologi Malaysia, Johor Bahru Johor, Malaysia; 2 College of Engineering Majmaah University, Majmaah, Kingdom of Saudi Arabia; National Institute of Genomic Medicine, Mexico

## Abstract

Closed form solutions for unsteady free convection flows of a second grade fluid near an isothermal vertical plate oscillating in its plane using the Laplace transform technique are established. Expressions for velocity and temperature are obtained and displayed graphically for different values of Prandtl number Pr, thermal Grashof number *Gr*, viscoelastic parameter *α*, phase angle *ωτ* and time *τ*. Numerical values of skin friction *τ*_0_ and Nusselt number Nu are shown in tables. Some well-known solutions in literature are reduced as the limiting cases of the present solutions.

## Introduction

It is well known that Newtonian fluids such as air, water, ethanol, benzene and mineral oils form a basis for classical fluid mechanics. However, many important fluids, such as blood, polymers, paint, and foods show non-Newtonian behavior. Due to the diversity of non-Newtonian fluids in nature no unique relationship is available in the literature that can describe the rheology of all the non-Newtonian fluids. Of course, the mathematical systems for non-Newtonian fluids are of higher order and complicated in comparison to the Newtonian fluids. Therefore, a variety of constitutive equations have been suggested to predict the behavior of non-Newtonian fluids. Despite of all these difficulties, the recent researchers in the field have made valuable contributions in study of flows of non-Newtonian fluids [1]–[12]. Amongst the different categorizations of non-Newtonian fluids, there is one simplest model of differential type fluids known as second grade fluid [13], [14]. Keeping the importance of non-Newtonian fluids in mind, for the present problem, we have chosen second grade fluid as a non-Newtonian fluid. Amongst the different studies on second grade fluids [15]–[25], Nazar et al. [26] provided some interesting results. They considered the second grade fluid over an oscillating plate and obtained exact solutions using the Laplace transform technique, expressed them as the sum of steady-state and transient solutions. Recently, Farhad et al. [27] extended the work of Nazar et al. [26] by considering the second grade fluid to be electrically conducting and passes through a porous medium. As a special case, it is observed that their results in the absence of MHD and porosity effects are reduced to those obtained by Nazar et al. [26].

On the other hand free convection is a common process in nature and has numerous applications and occurrences in industry. It is a major cause of atmospheric and oceanic circulation and plays an important role in the passive emergency cooling systems of advanced nuclear reactors. Furthermore, free convection flows of non-Newtonian fluids with heat transfer play an important role in many industrial systems. For example, there are many process in which thermal energy is transferred from an object through the physical contact with heat transfer fluids at a temperature colder than the object. Industrial refrigeration or heating, chemical manufacturing, breweries, ventilation and air conditioning, ice rinks and engine cooling, environmental chambers, oil and gas industry and, food and pharmaceutical are some examples of such applications [28]–[30]. Besides that, the Stokes’ second problem for the flow of an incompressible fluid over an oscillating plane is of great importance in the literature of fluid dynamics. It admits an exact analytical solution [31]. The Stokes’ or Rayleigh problem is not only of fundamental theoretical interest but it also occurs in many applied problems [32], [33].

Pop and Watanabe [34] investigated the effects of suction and injection on the free convection flow from vertical cone with uniform surface heat flux with fixed value of Pr = 0.7 and obtained numerical solutions. Kafoussias [35] studied free convection magnetohydrodynamic flows through porous medium and obtained numerical solutions for constant viscosity. In the investigations [34], [35], the coefficients of viscosity are assumed constant. However, it is observed the coefficients of viscosity for most fluids may depend on temperature [36]. Many investigations have been reported into the problem of free convection heat transfer along a vertical surface with temperature dependent viscosity for different heating conditions [37]–[41]. Jang and Lin [42] studied the role of temperature-dependent viscosity in laminar free convection flow adjacent to a vertical surface with uniform heat flux.

Most of the existing studies in the literature on convection flows of second grade fluid are concerned with numerical or approximate solutions [43]–[45]. Considerably less work has been reported concerning the constant property effects on free convection flow of second grade fluid over the vertical isothermal plate. So, it is necessary to carry out the study on free convection flows of second grade fluid with exact solutions for the free convection flow of second grade. Exact solutions on the other hand are needed not only for the technical relevance of the flows but are also significant for a variety of other reasons such as they can be used as a benchmark by numerical solvers and for checking the stability of their solutions. Therefore, the main purpose of the present investigation is to study the unsteady free convection flow of a second grade fluid past an isothermal vertical plate oscillating in its plane with constant viscosity [34], [35], and to obtain the exact solutions using the Laplace transform technique. The present problem is the extension of Nazar et al. [26]. However, it is rather complicated due to the presence of free convection term in the momentum equation which makes the momentum and energy equations coupled with each others. Hence the present solutions are more general compared to the solutions existing in the literature.

## Formulation of the Problem

Following Fosdick and Rajagopal [13], the Cauchy stress tensor 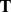 in a homogeneous incompressible fluid of second grade is related to the fluid motion in the following form
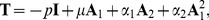
(1)where 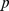 is the scalar pressure, 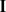 is the identity tensor, 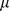 is the coefficient of viscosity, 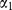 and 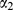 are the material moduli commonly referred to as the normal stress moduli and 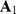 and 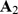 stand for the first two tensor of Rivlin and Ericksen defined by
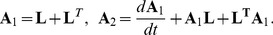
(2)


According to Fosdick and Rajagopal [13] and Dunn and Fosdick [14] the model (1) required to be compatible with thermodynamics in the sense that all motions satisfy the Clausius-Duhem inequality and the assumption that the specific Helmholtz free energy is a minimum in equilibrium at constant temperature then, the material moduli must satisfy the following conditions
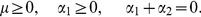
(3)


**Figure 1:**
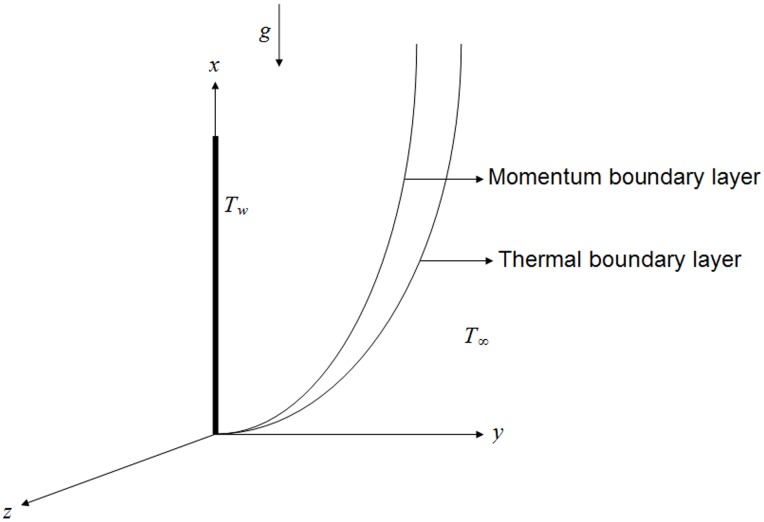
Physical geometry and coordinates system.

Now let us consider the unsteady free convection flow of a second grade fluid near an isothermal vertical plate situated in the 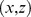 plane of a Cartesian coordinate system 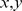 and 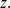 Initially, both the plate and fluid are at rest with constant temperature 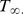 At time 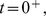 the plate starts motion in its plane with oscillating velocity and then transmitted to the fluid. The temperature of the plate immediately raises to 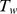 and thereafter maintains constant. Owing to the shear, the fluid is gradually moved and its velocity is of the form
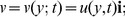
(4)where 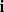 is the unit vector in the flow direction as shown in Fig. 1.

In the view of the above assumptions and using the usual Boussinesq approximation, the momentum and energy equations for the incompressible flow of a second grade fluid are
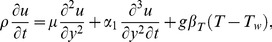
(5)

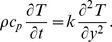
(6)


The appropriate initial and boundary conditions
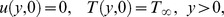


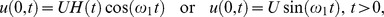


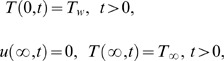
(7)where 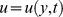 denotes the fluid velocity in the 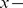
direction, 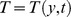 is the temperature, 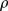 is the constant density of the fluid, 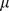 is the viscosity, 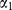 is the second grade parameter, 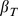 is the volumetric coefficient of thermal expansion, 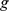 is the acceleration due to gravity, 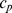 is the specific heat capacity, 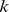 is the thermal conductivity, 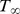 is the free stream temperature, 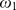 the frequency of the velocity of the wall and 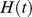 is the Heaviside unit step function.

By introducing the following dimensionless variables
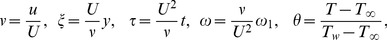
(8)the system of equations 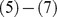 reduces to



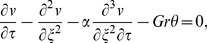
(9)

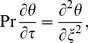
(10)

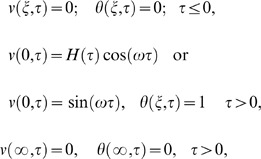
(11)where



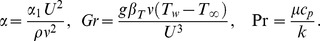



Here 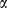 is the dimensionless second grade parameter, 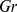 is the thermal Grashof number and 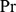 is the Prandtl number.

## Solution of the Problem

We solve the governing equations in exact form by the Laplace transform technique and their solutions in the transform 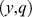-plane are given by
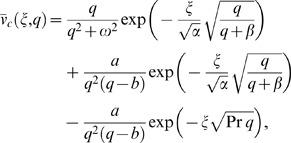
(12)

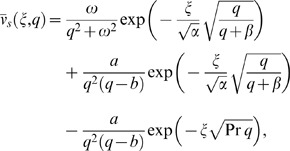
(13)

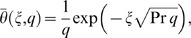
(14)where the subscripts 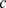 and 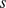 in Eqs. (12) and (13) refer to cosine and sine oscillations of the plate and



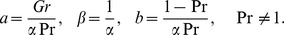



We split Eq. (12) in the following forms
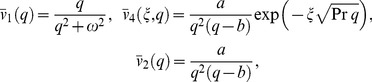
(15)

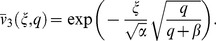
(16)


Let us we denote
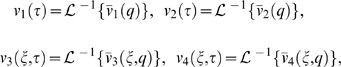
where 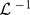 is denoting the inverse Laplace transform.

In order to find the inverse Laplace transform of Eq. (12), we write the velocity 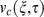 as a convolution product (see theorem (A1) from Appendix S1).
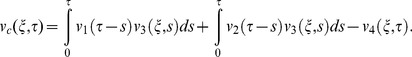
(17)


Laplace inversion of Eq. 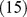 leads to the following expressions.
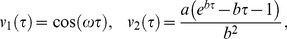
(18)

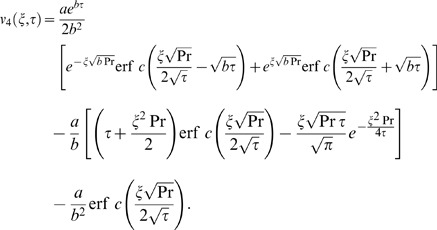
(19)


In order to find 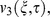
we use the inversion formula of compound functions (A2) and some of the known results (A4)-(A7) from Appendix S1, consequently Eq. (16) results.
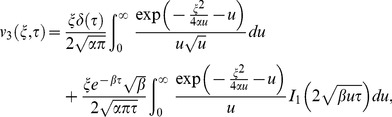
(20)where 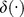 is the Dirac delta function and 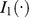 is the modified Bessel function of the first kind of order one. Using Eqs. (18)–(20) into Eq. (17), keeping in mind (A3) from Appendix S1, we get
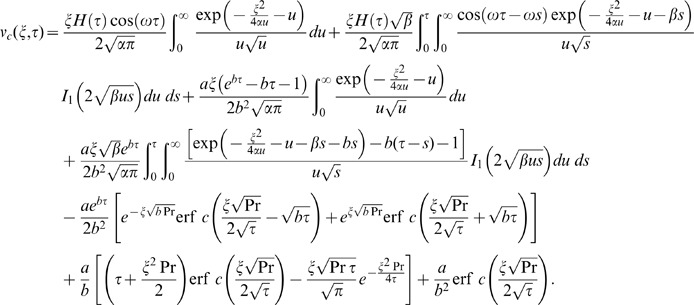
(21)


Similarly for the sine oscillations of the plate the corresponding expression of velocity is given by
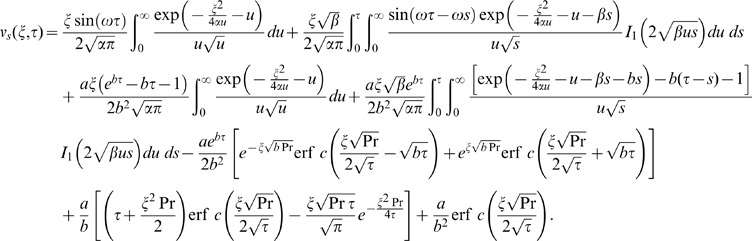
(22)


The starting solutions of 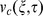 and 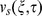
given by Eqs. (21) and (22) are rather complicated. Therefore, we derive approximate expressions for these velocities corresponding to small and large values of time. This time is important, especially for those who need to eliminate transients from their rheological measurements [26]. In order to determine this time, we need first to write the starting solutions as the sum of the steady state and transient solutions. Therefore, we decompose the integrals from Eqs. (21) and (22) under the form
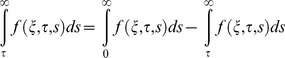
(23)and use formulae (A8)-(A10) from Appendix S1, we obtain
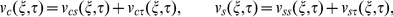
(24)where the steady state solutions are written as
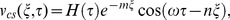
(25)

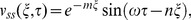
(26)which are periodic in time and independent of the initial condition. The transient solutions in equivalent but more suitable forms are written as
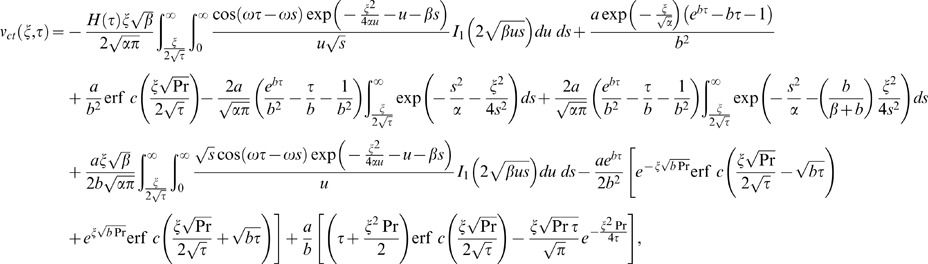
(27)

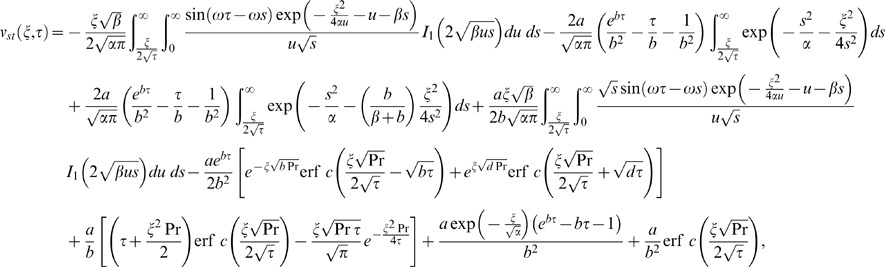
(28)in which
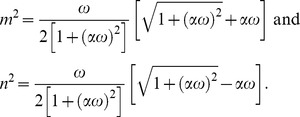



The inverse Laplace transform of Eq. (14) gives the required temperature as
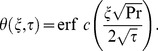
(29)


It is important to note that the steady state solutions (25) and (26) are independent of thermal effects whereas, the transient solutions (27) and (28) contain the thermal effects due to the presence of free convection term. Therefore, these transient solutions can be written as a sum of the mechanical 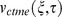 and thermal 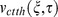 components as below
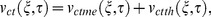
(30)

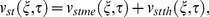
(31)where



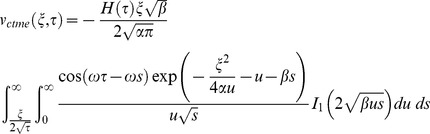
(32)

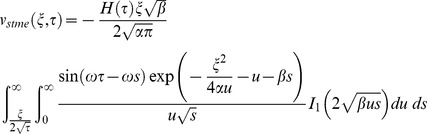
(33)

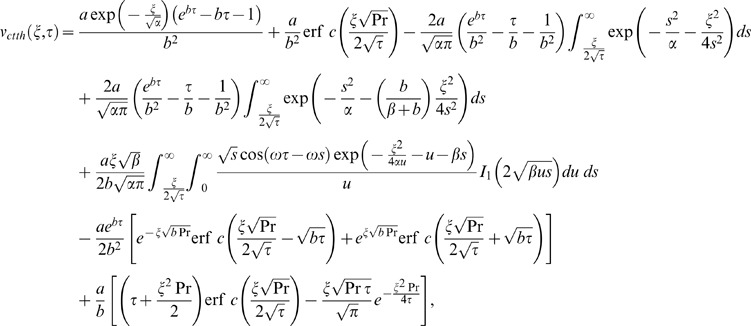
(34)

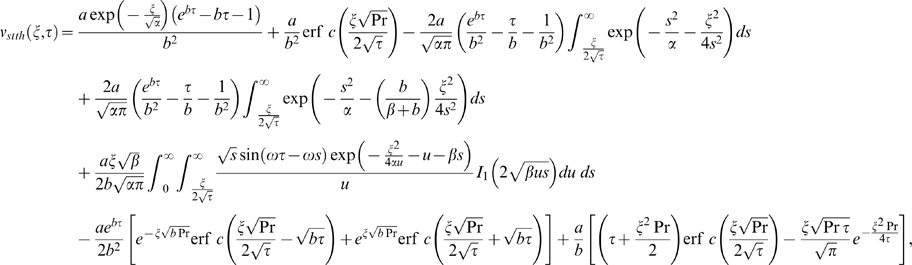
(35)in which the subscripts *me* and *th* are used for the mechanical and thermal parts of transient velocity.

Further, it is worth mentioning to note that solutions (21) and (22) are valid only for 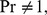 however to make these solutions valid for 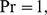 we once again derive our solutions by putting 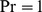 into Eq. (14) and using it in the transform solution of Eq. (9), the starting solutions are
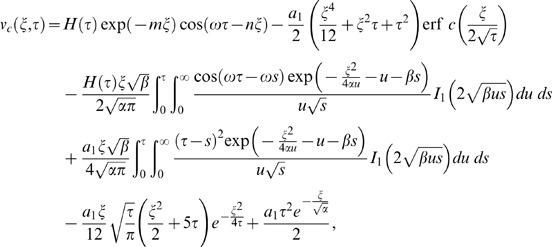
(36)

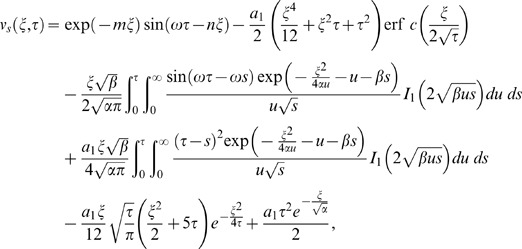
(37)corresponding to the cosine and sine oscillations of the plate and 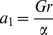. Now by employing the previous methodology, the starting solutions (36) and (37) can also be written as a sum of the steady-state and transient solutions.

## Limiting Cases

Equations (21) and (22) investigate the exact solutions for the starting motion of a second grade fluid for the cosine and sine oscillations of an isothermal vertical plate respectively and Eq. (28) represents the corresponding solution for temperature of the fluid. Since the present solutions are more general and the existing published results from the literature appear as special cases by taking suitable parameters such as Grashof number 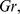 frequency of oscillations 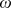 and the second grade parameter 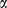 equal to zero.

### Case-I: Solutions in the Absence of Thermal Effects

In the absence of free convection, the solution of temperature (29), is unaffected by the thermal effects due to the reason that the free convection term 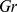 is not involved there, however by taking 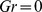 implies that 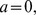 Eqs. 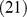 and 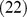 yield Eqs. 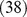 and 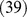 as follows
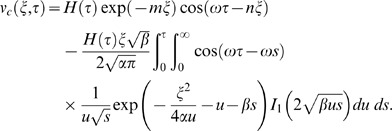
(38)

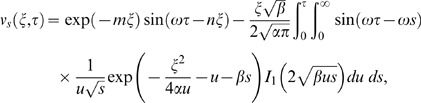
(39)which are identical to the starting solutions obtained by Nazar et al. (Eqs. (13) and (14) in [26]) describe the motion of the fluid for small and large times. Furthermore, for 
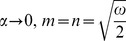
, the steady parts of Eqs. 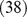 and 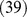 give the well known results



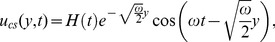
(40)

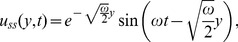
(41)which are quite identical to the published results obtained by Erdogan (Eqs. 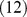 and 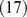 in [46]) and Feteca et al. (Eq. 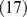 in [47]).

### Case-II: Solutions in the Absence of Oscillating Effects

Now let us assume that the infinite plate is set into impulsive motion after time 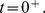
The thermal component of velocity 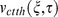 remain unchanged while the mechanical part of velocity 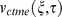 is effected due to the frequency of oscillations 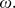 So, by taking 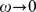 into Eq. 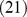, the solution corresponding to the case when the plate applies impulsive motion to the fluid is given by
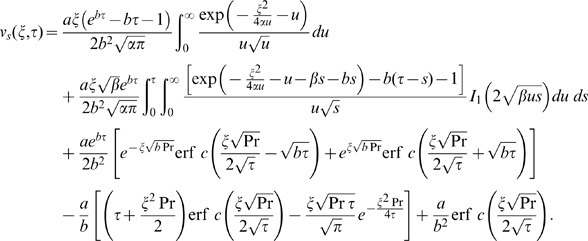
(42)


### Case-III: Solutions in the Absence of Mechanical Effects

Here we assume that the infinite plate is kept at rest all the time. In this case the motion in the fluid together with heat transfer are only caused due to the presence of free convection because there is no disturbance from the bounding plate. Thus, the mechanical component of velocity is identically zero and consequently the velocity of the fluid 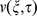 reduces to the thermal component
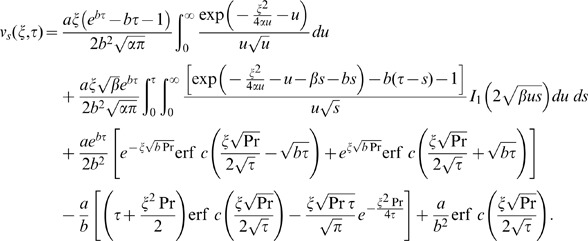
(43)


We note that the solutions obtained as limiting cases (Case-II & Case-III) are also new and not available in the literature.

### Skin-Friction

The expression for dimensional skin friction in case of a second grade fluid is given as
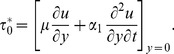
(44)


In dimensionless form Eq. 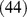 is written as
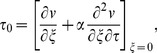
(45)where 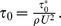


Finally, Eq. 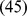 in view of Eq. 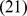 gives
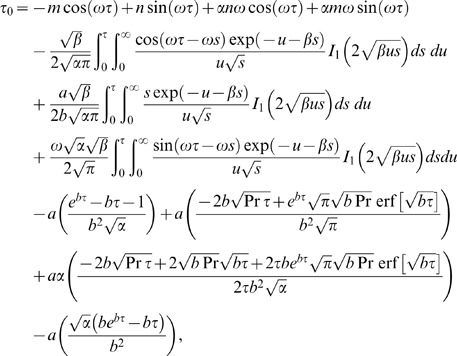
(46)


### Nusselt Number

The rate of heat transfer evaluated from Eq. 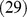 is given by
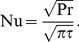
(47)


## Results and Discussion

A numerical assessment for the exact solutions 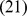 of the present problem corresponding to the cosine oscillations of the plate and 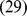 is performed. Using a computational software Mathcad, the results are plotted to illustrate the interesting features of the involved parameters on the starting solution corresponding to the cosine oscillations of the plate (Figs. 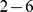 ) and temperature profiles (Fig. 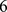 and 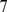) whereas Figs. 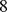 and 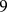 are shown for the starting and steady-state velocities corresponding to the cosine and sine oscillations of the plate. In addition Fig. 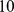 is prepared to show the comparison of the present results with Nazar et al. 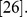
The parameters entering into the problem are second grade parameter 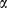, Prandtl number 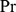, thermal Grashof number 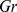, dimensionless time 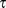, and phase angle 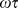.

**Figure 2:**
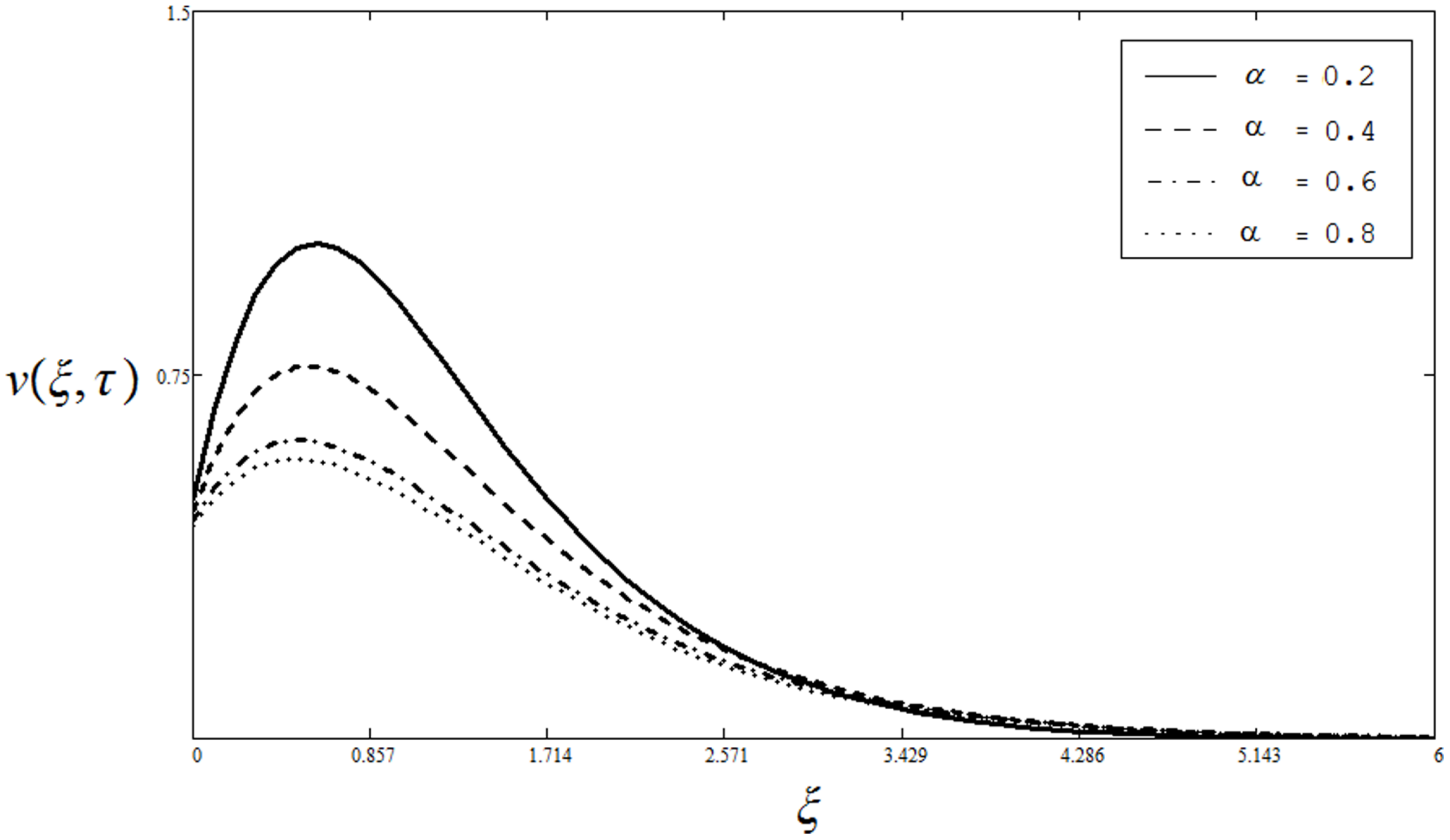
Velocity profiles for different values of 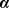 when 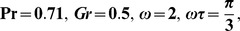
and 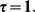

Figure 2 shows the influence of 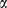 on the velocity field 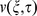. It is clear from this figure that an increase in 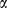 results a decrease in the velocity. Physically, it is true because the higher values of 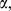 are having greater stability than the smaller values. This behavior of 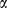 is quite similar to that of Sivaraj and Kumar (see Fig. 4 in [32]). Unlike [34], [35], the effect of Prandtl number 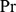 for four different values as 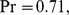 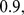 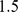 and 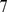 upon velocity 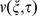 is elucidated from Fig. 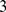. It is seen from this figure. 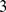 that in the case of heating of the plate or cooling of the fluid 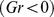, velocity 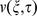 decreases when Prandtl number 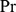 increases. Physically, it is true as the Prandtl number describes the ratio between momentum diffusivity and thermal diffusivity and hence controls the relative thickness of the momentum and thermal boundary layers. As 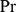 increases the viscous forces (momentum diffusivity) dominate the thermal diffusivity and consequently decreases the velocity. The influence of thermal Grashof number 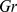 on velocity distribution 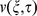 is elucidated from Fig. 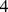. It is clear from this figure that in the absence of thermal effect 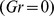 when the effect of buoyant forces is negligible and the viscous forces are dominant, the velocity tends to steady-state faster than for the values of 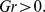 It can be observed that velocity increases for the increasing values of 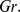 It is also true physically as the Grashof number 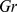 describes the ratio of bouncy forces to viscous forces. Therefore, an increase in the values of 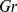 leads to increase in buoyancy forces, consequently velocity increases.

**Figure 3:**
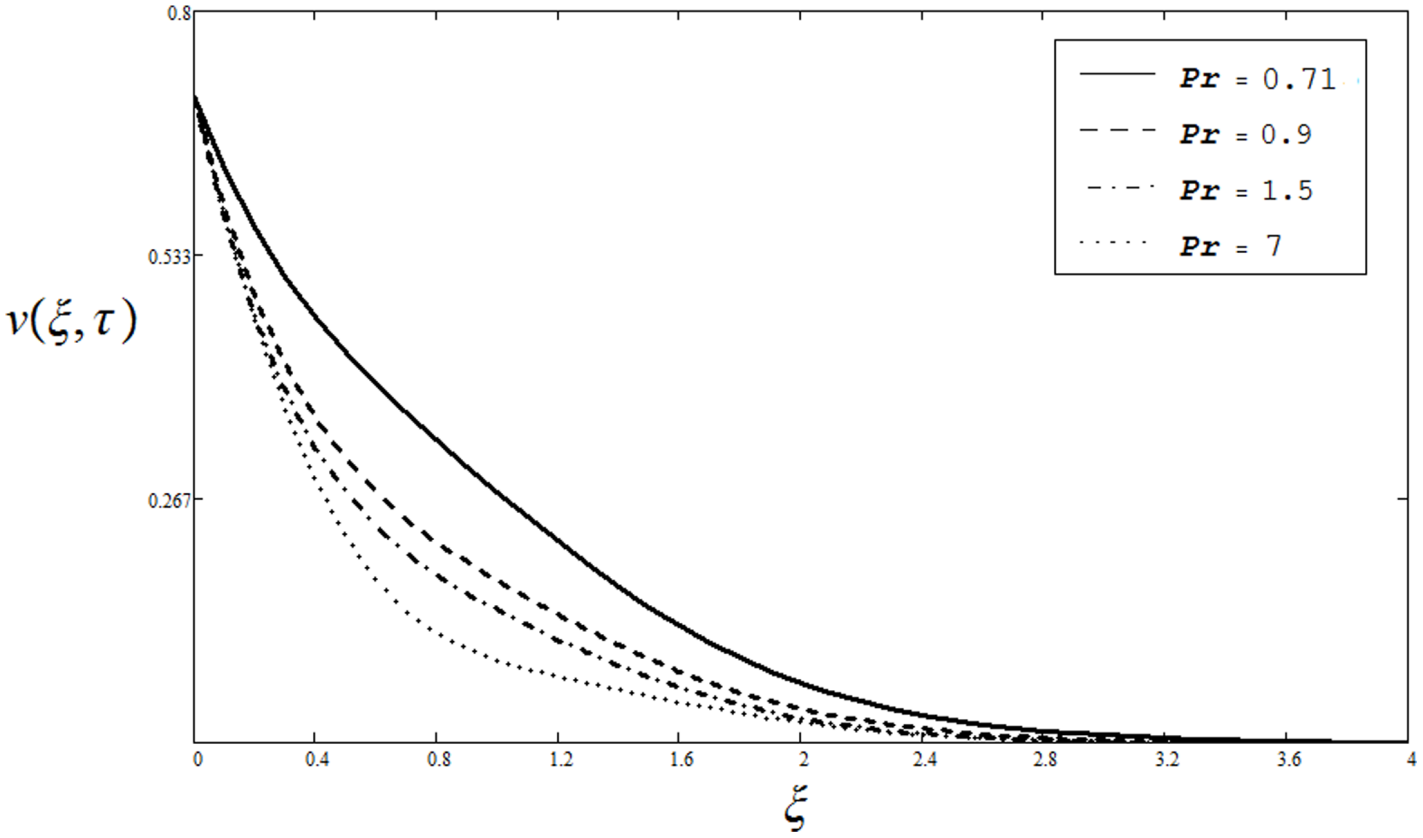
Velocity profiles for different values of 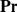 when 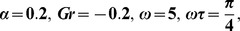
and 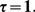

**Figure 4:**
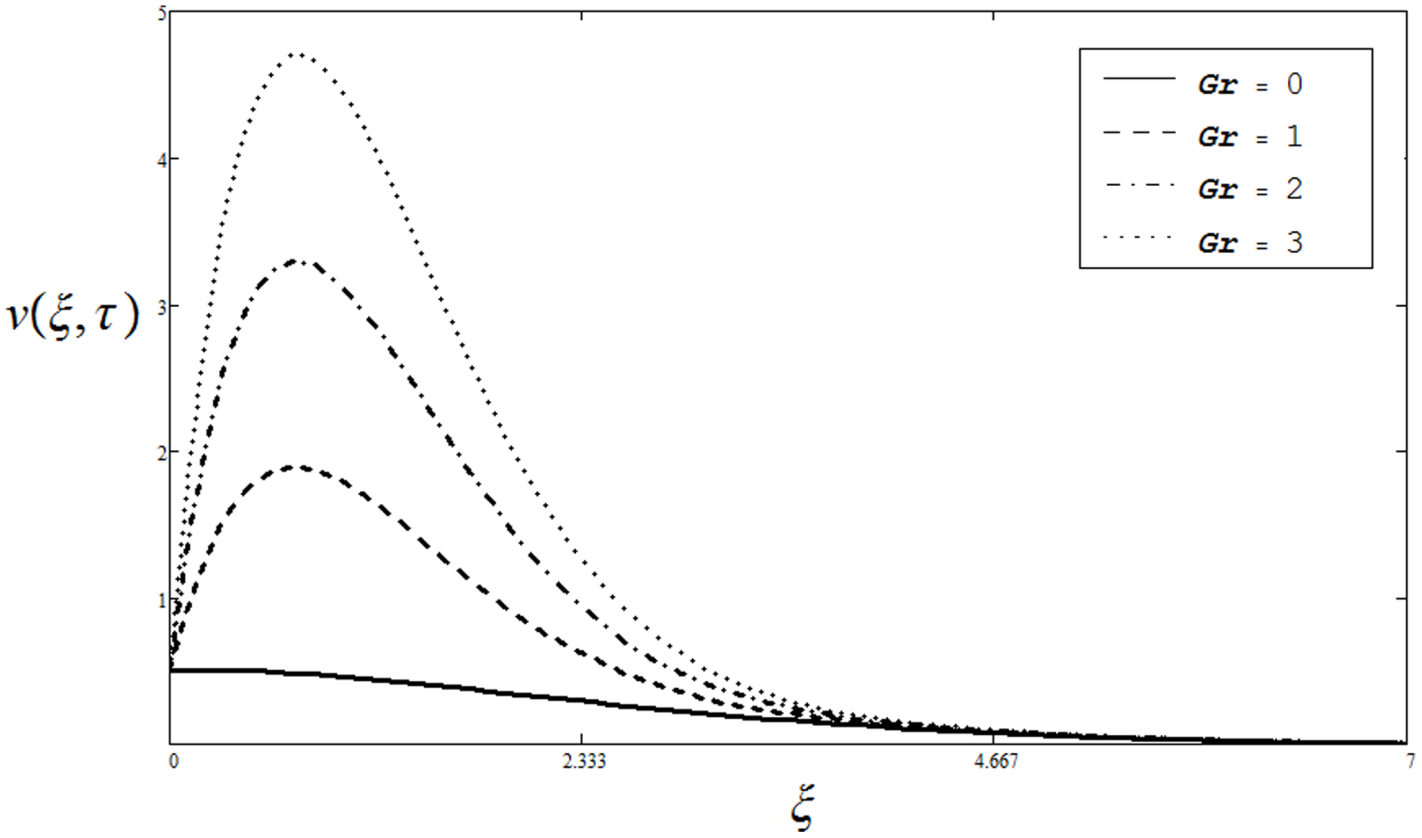
Velocity profiles for different values of 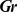 when 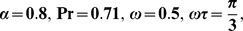
and 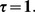

**Figure 5:**
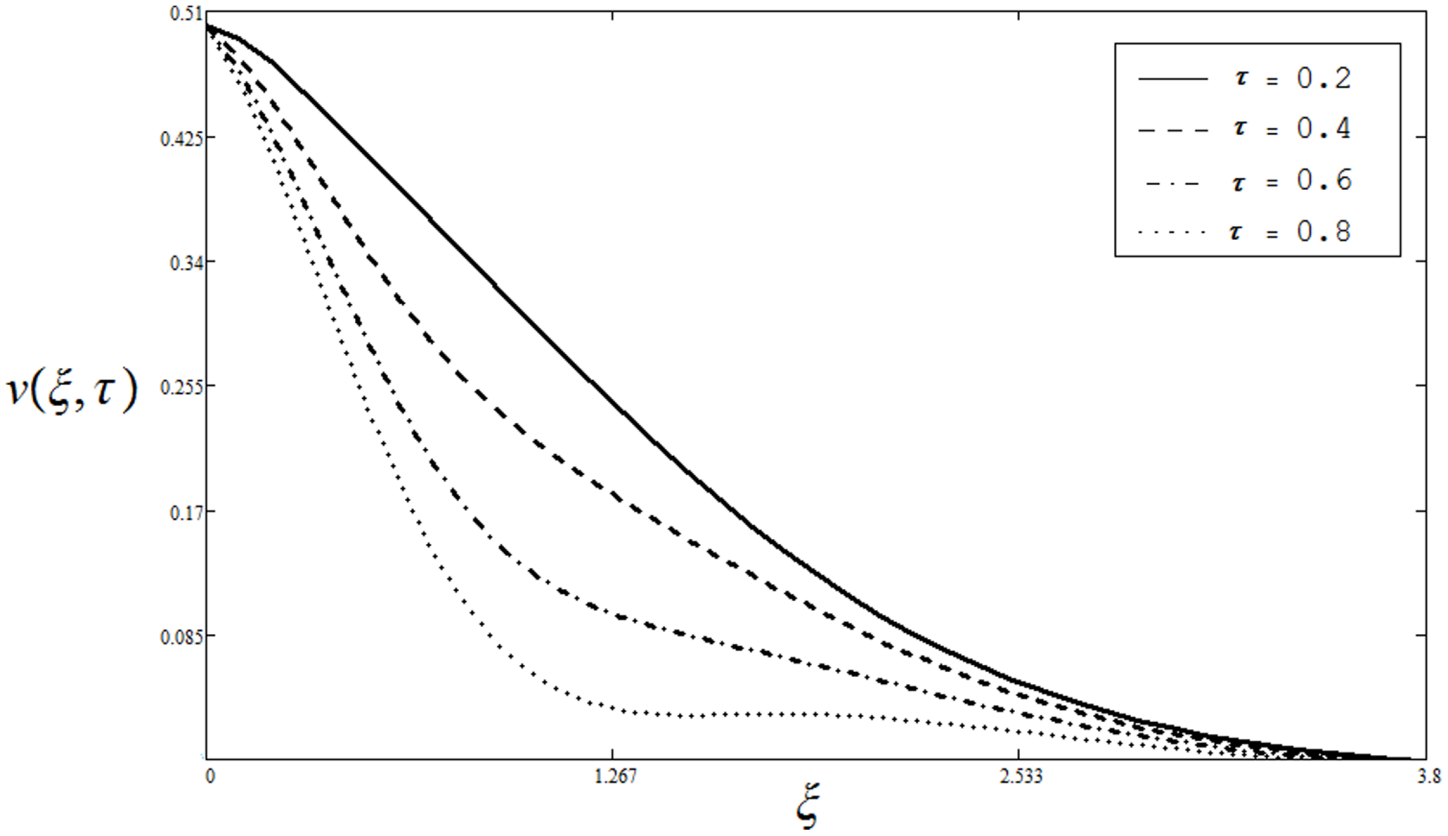
Velocity profiles for different values of 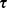 when 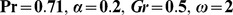 and 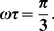

**Figure 6:**
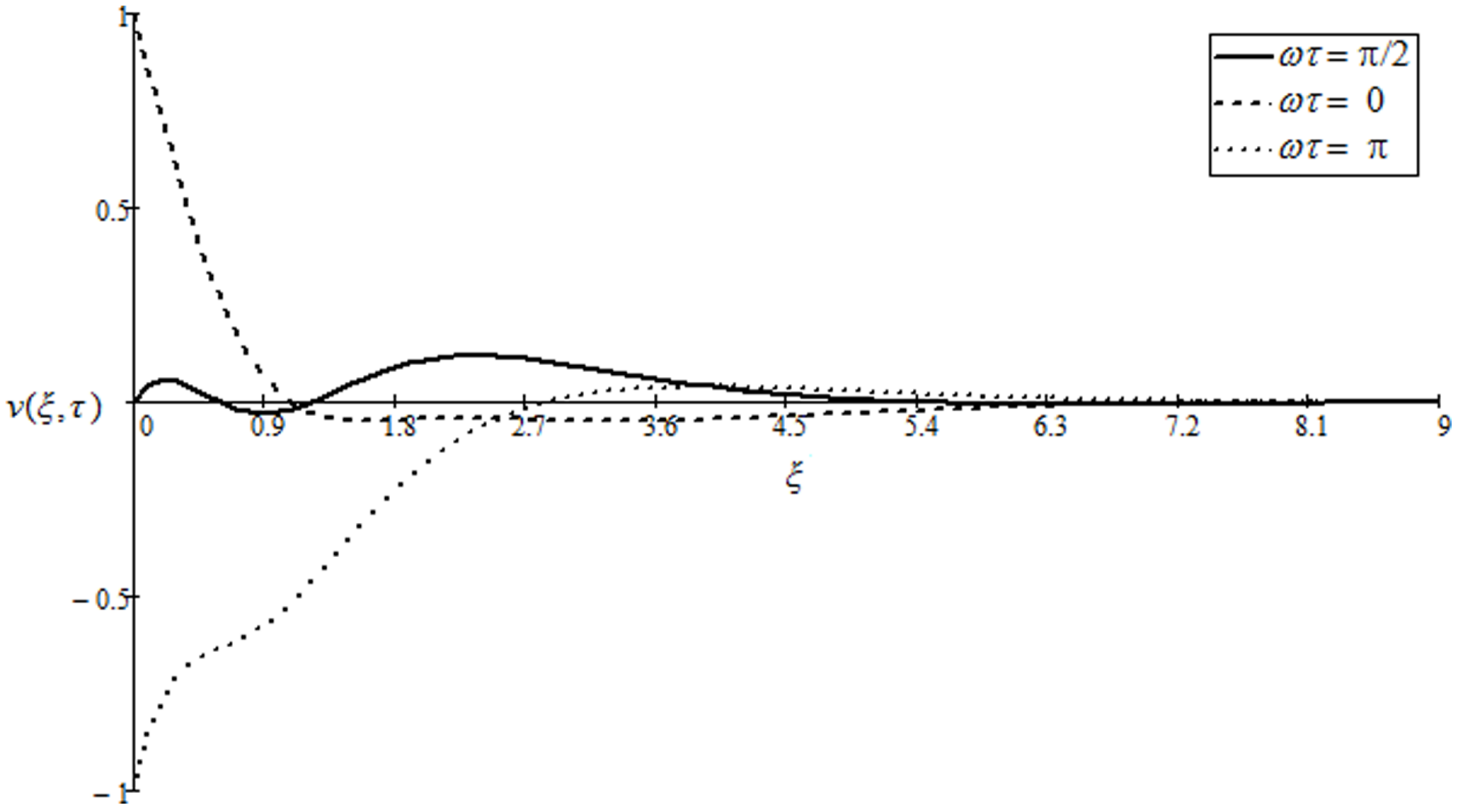
Velocity profiles for different values of 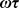 when 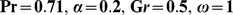
and 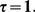

**Figure 7:**
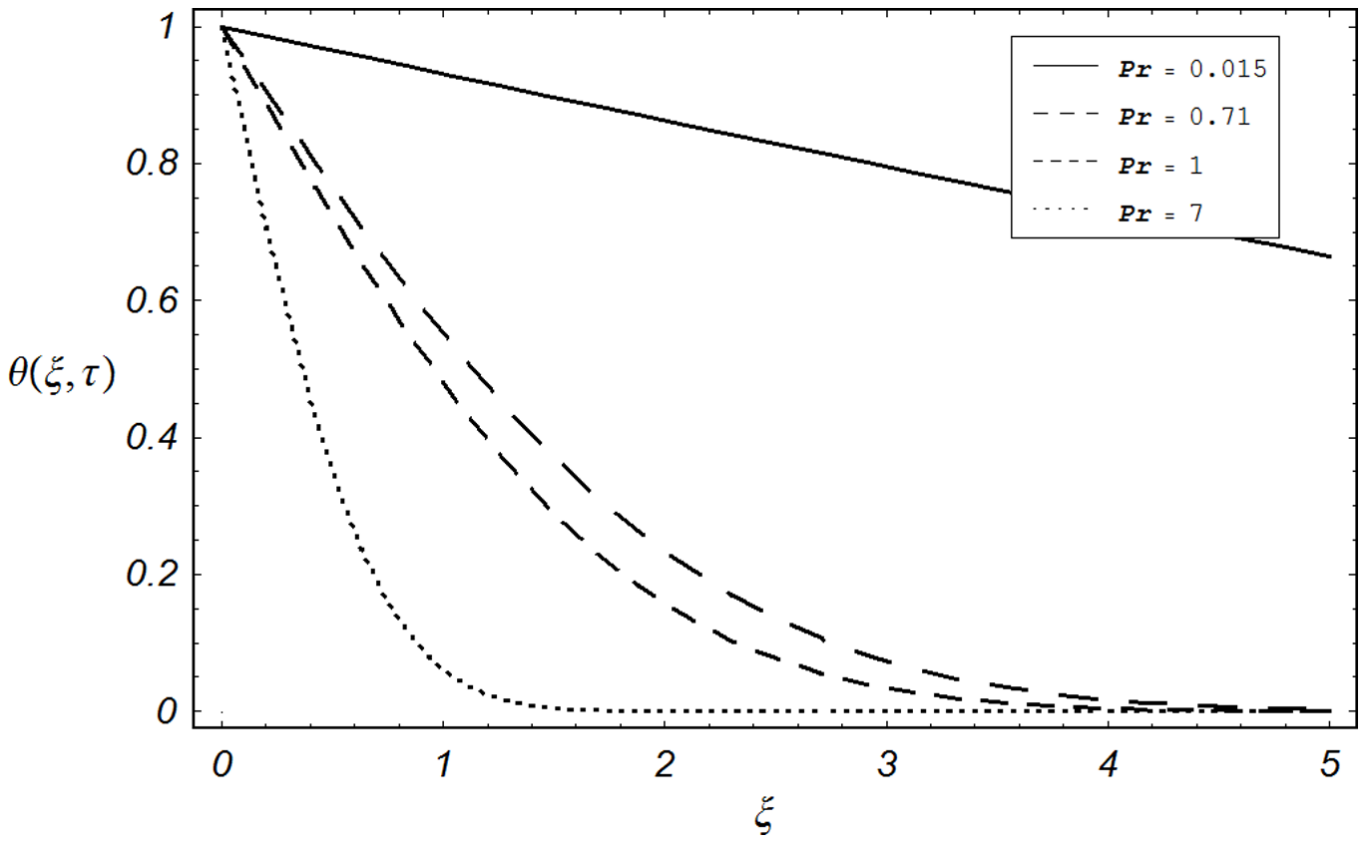
Temperature profiles for different values of 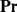 when 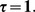

**Figure 8:**
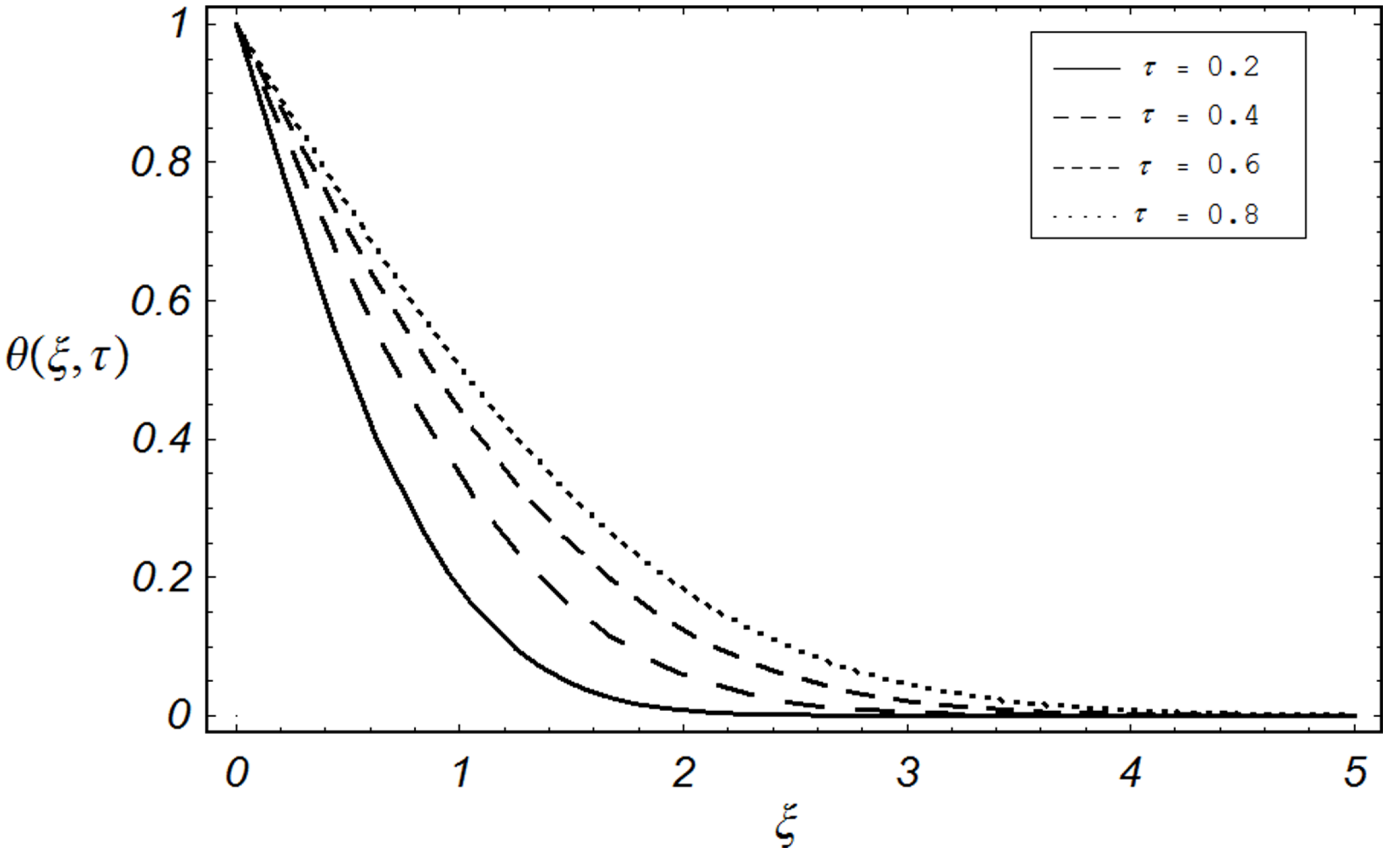
Temperature profiles for different values of 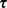 when 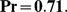

The effect of dimensionless time 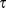 on velocity 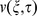 is illustrated from Fig. 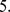 It can be seen from this figure that velocity is a decreasing function of 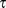. The effect of phase angle 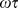 upon velocity 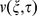 is elucidated from Fig. 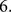 It is observed that velocity 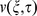 is fluctuating between −1 and 1, tending to zero for large values of independent variable 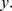 It is clear from this figure that the obtained solution (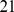) satisfies the corresponding boundary conditions given in Eq. (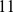). Hence this provides a useful mathematical check. The influence of Prandtl number 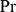 on temperature profile 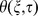 is shown in Fig. 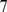. Four different values of 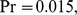 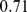, 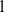 and 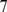 are chosen. They physically correspond to mercury, electrolyte, air and water respectively. It is found that temperature decreases when 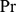 is increased. As 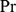 is the ratio of momentum diffusivity (kinematic viscosity) to that of thermal diffusivity, so the increase in 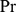 is actually increase in viscous forces (viscosity) which results a decrease in temperature profile. The effect of dimensionless time 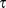 on the temperature profiles 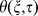 is shown in Fig.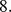 It can be seen from the figure that the effect of time 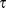 on temperature 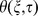 is quite opposite to the Prandtl number 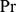 as observed in Fig. 7.

**Figure 9:**
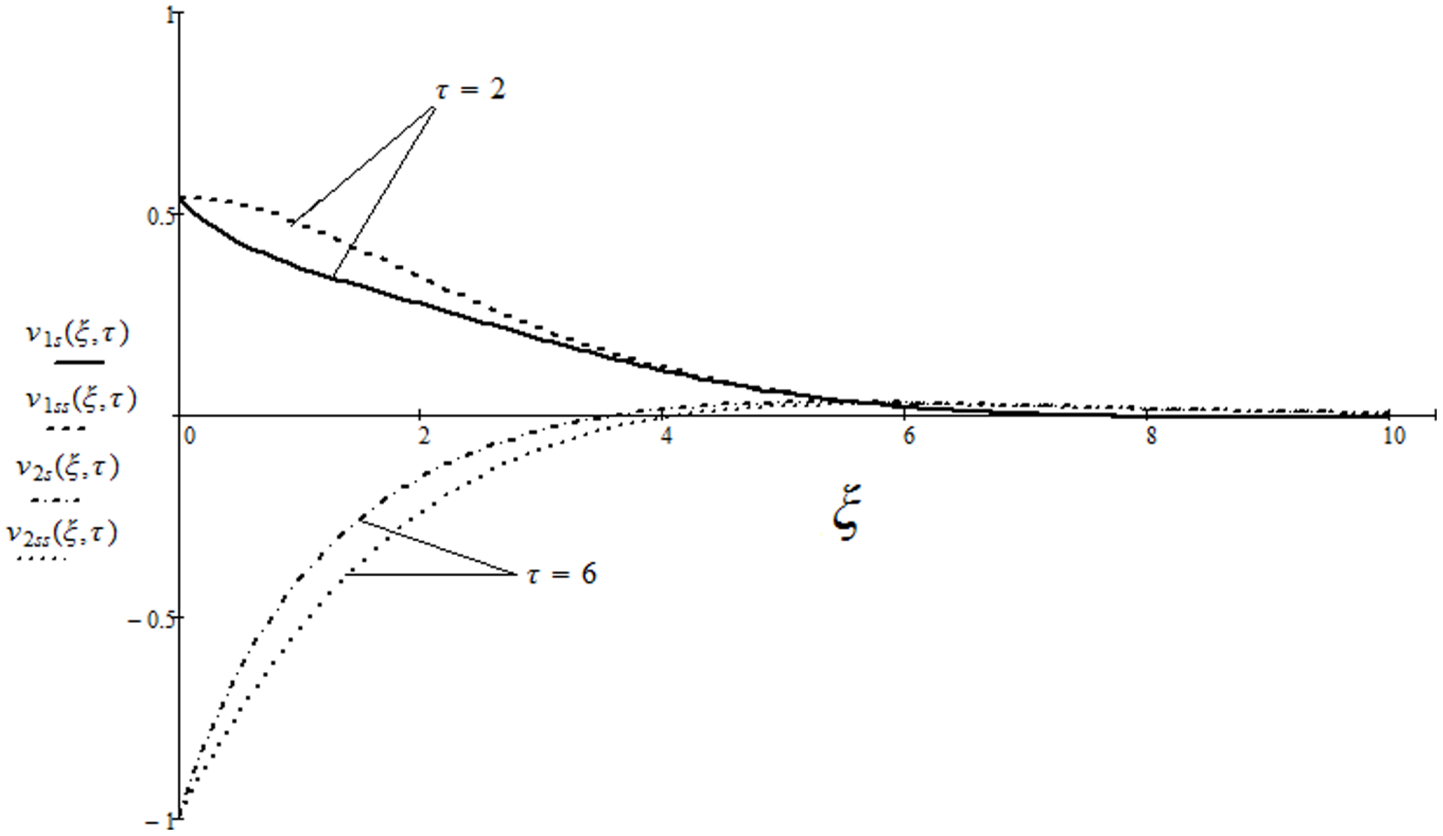
Variations of the starting and steady-state solutions with the distance from the wall, for the cosine oscillations of the boundary, corresponding to relation (21) curves 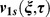, 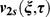
and relation (25) curves 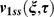, 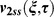, when 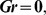 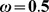 and 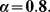

**Figure 10:**
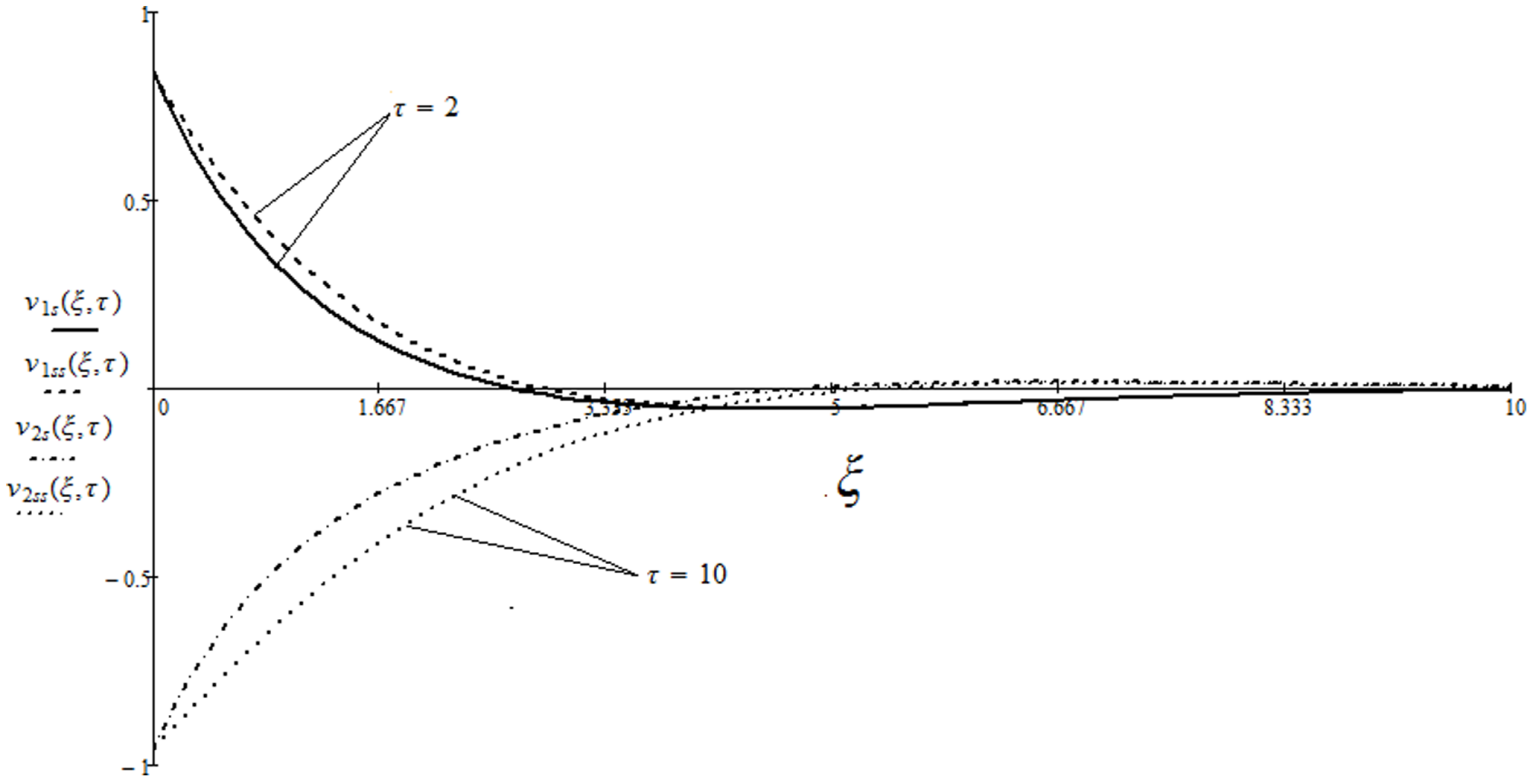
Variations of the starting and steady-state solutions with the distance from the wall, for the sine oscillations of the boundary, corresponding to relation (22) curves 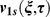, 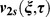
and relation (26) curves 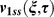, 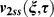, when 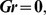 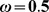 and 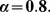

A very important problem regarding the technical applicability of the starting solutions is to find the approximate time after which the fluid is moving according to the steady-state solutions. More exactly, in practice it is necessary to know the required time to attain the steady state [26]. For this purpose, the variations of the corresponding starting and steady-state velocities with the distance from the wall are depicted in Figs. 9 and 10. At small values of time, the difference between unsteady and steady-state velocities is large enough. This difference rapidly decreases and it can be clearly seen from the figures that the required time 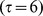 to reach the steady-state for the cosine oscillations of the boundary is smaller than that for the sine oscillations 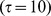. A comparative study of the present solution 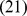 corresponding to the cosine oscillations of the plate is provided in Fig. 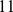 with published results of Nazar et al. (Eq. (13) in [26]) It is found that in the absence of free convection (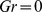) the present results are identical with those of Nazar et al. [26].

**Figure 11:**
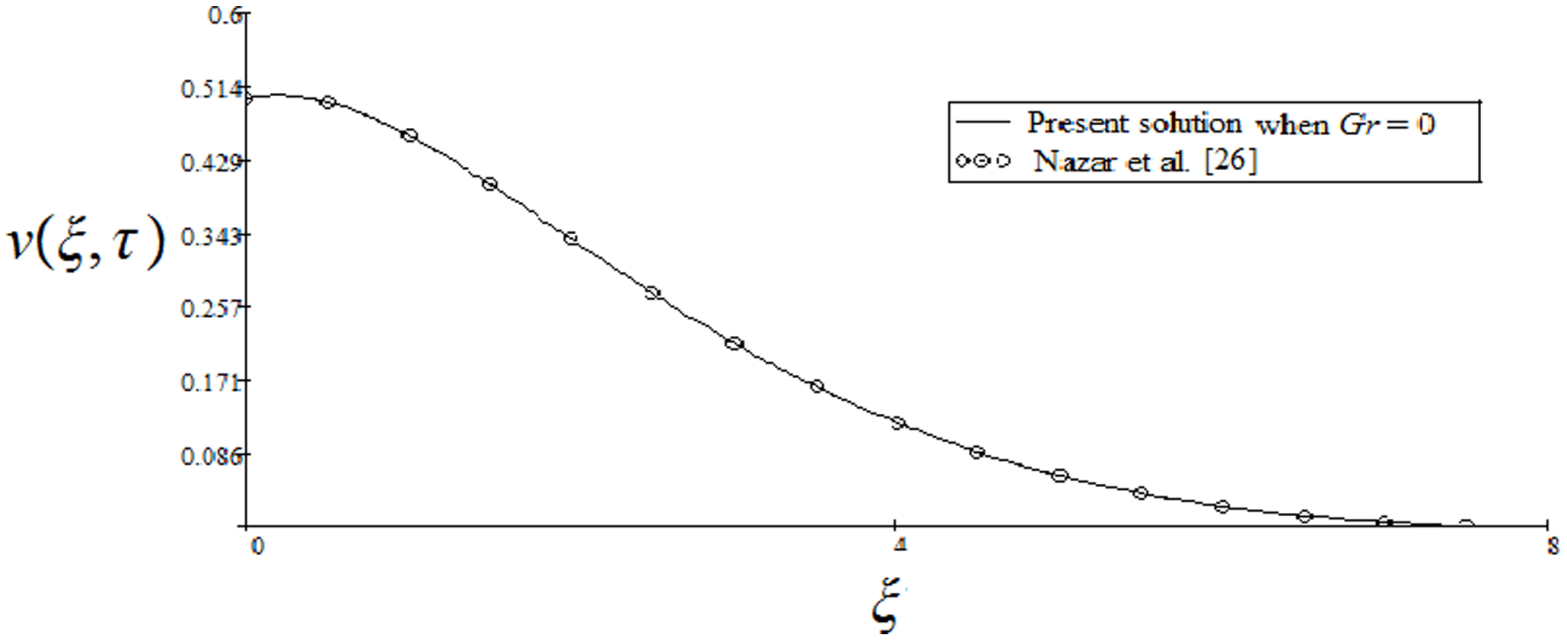
Comparative study of the present solution 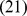 to those of Nazar et al. (Eq. (13) in [26]) corresponding to the >cosine oscillations of the plate when 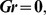 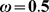, 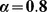 and 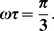

The numerical results for skin friction 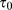 are shown in Table 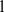
for various embedded parameters. It is found that the skin friction decreases when 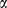 is increased. On the other hand, the influence of Prandtl number 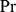 on skin friction shows that 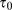
decreases when 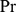 increases whereas it increases for large values of 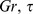 and 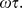 The effects of 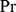 and 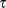 on Nusselt number Nu are studied numerically in Table 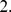 It is found that Nu

decreases when 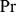 increases. Physically this behavior is acceptable because when 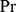 increases, it decreases the resistance and consequently enhances the rate of heat transfer. The influence of 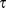 on Nu is found quite opposite to that of 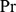.

**Table 1:** Variation in skin-frictions 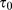

α	P_r_	G_r_	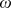	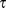	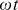	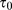
0.2	0.71	0.5	0.5	1	π/3	7.137
0.2	0.71	0.5	0.5	1	π/3	2.150
0.2	**0.9**	**1**	0.5	1	π/3	14.728
**0.4**	0.71	0.5	0.5	1	π/3	1.203
0.2	0.71	0.5	**0.8**	1	π/3	7.194
0.2	0.71	0.5	0.5	**2**	π/3	32.00
0.2	0.71	0.5	0.5	2	π	9.199

**Table 2:** Variation in Nusselt number Nu.

P_r_	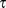	N_u_
0.71	1	0.47
**7**	1	1.492
0.71	**2**	0.33

## Conclusions

The heat transfer analysis of a second grade fluid for unsteady free convection flow past an isothermal vertical plate oscillating in its plane is investigated. Closed form solutions of the problem are obtained by using the Laplace transform technique. The starting solutions (21) and (22) are expressed in terms of steady-state and transient solutions. It is found that they satisfy the imposed initial and boundary conditions and can be easily reduced to the similar solutions in the literature by taking Grashof number 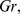 frequency of oscillations 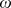 and the second grade parameter 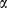 equal to zero. The effects of various parameters on velocity and temperature profiles are graphically studied whereas the results for skin-friction and Nusselt number are computed in tables. The following conclusions are extracted from this study.

- Increasing second grade parameter 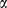 decreases fluid velocity.
- Velocity for electrolyte solution is greater than air and water.
- The presence of free convection enhances the fluid motion.
- Temperature decreases for large values of 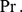

- The Nusselt number increases when 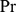 is increased
- The skin friction increases when both time 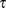 and phase angle 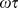 are increased.
- In the absence of free convection (Gr = 0) the present solutions are found identical to those obtained by Nazar et al. [26].

## Supporting Information

Appendix S1 (PDF)
