# Supplementary material for: Closed Form Solutions for Unsteady Free Convection Flow of a Second Grade Fluid over an Oscillating Vertical Plate
Source: PLoS One. 2014 Feb 14;9(2):e85099. doi: 10.1371/journal.pone.0085099 (PMC3925078; doi:10.1371/journal.pone.0085099)
Supplement: Appendix S1 — (PDF) [file pone.0085099.s001.pdf]

Supporting Information

Appendix S1

Convolution theorem

If $F(q)$, $G(q)$ and $H(q)$ are the Laplace transforms of the functions $f(\tau)$, $g(\tau)$, $h(\tau)$ respectively, and $H(q) = F(q)\,G(q)$, then

$$h(\tau) = (f * g)(\tau) = \int_0^\tau f(s)\,g(\tau - s)\,ds = \int_0^\tau f(\tau - s)\,g(s)\,ds \tag{A1}$$

where the symbol " $*$ " denotes the convolution product of the two function.

Inversion formula for compound functions

if $f(\tau) = \mathcal{L}^{-1}\{F(q)\}$ and $g(u,\tau) = \mathcal{L}^{-1}\left\{e^{-uw(q)}\right\}$, then the inverse Laplace transform of the compound function $F[w(q)]$ is

$$\mathcal{L}^{-1}\{F[w(q)]\} = \int_0^\infty f(u)\,g(u,\tau)\,du. \tag{A2}$$

If $F(q)$ is the Laplace transform of the function $f(\tau)$ and the Dirac delta function $\delta(\cdot)$ is the unit element for the convolution product, i.e.

$$(\delta * f)(\tau) = (f * \delta)(\tau) = f(\tau), \quad \text{for each function } f(\cdot). \tag{A3}$$

$$\mathcal{L}^{-1}\{1\} = \delta(\tau); \quad \mathcal{L}^{-1}\left\{\frac{1}{q^{n+1}}\right\} = \frac{\tau^n}{n!}; \quad n = 0, 1, 2, ... \tag{A4}$$

$$\mathcal{L}^{-1}\left\{\frac{1}{q^2 + a^2}\right\} = \frac{1}{a}\sin(a\tau), \quad a \neq 0, \quad \mathcal{L}^{-1}\left\{\frac{q}{q^2 + a^2}\right\} = \cos(a\tau). \tag{A5}$$

$$\mathcal{L}^{-1}\{\exp(a\sqrt{q})\} = \frac{a}{2\tau\sqrt{\pi\tau}}\exp\left(-\frac{a^2}{4\tau}\right); \quad \mathrm{Re}\left(a^2\right) > 0. \tag{A6}$$

$$\mathcal{L}^{-1}\left\{\exp\left(\frac{\beta u}{q + \beta}\right) - 1\right\} = \sqrt{\frac{\beta u}{\tau}}\exp(-\beta\tau)\,I_1\left(2\sqrt{\beta u\tau}\right). \tag{A7}$$

$$\int_0^\infty \exp\left(-a^2 z^2 - \frac{b^2}{z^2}\right)dz = \frac{\sqrt{\pi}\exp(-ab)}{2a}, \quad \mathrm{Re}\left(a^2\right) > 0, \ \mathrm{Re}\left(b^2\right) \geq 0, \ \mathrm{Re}(ab) > 0. \tag{A8}$$

$$\int_0^\infty \int_0^\infty \frac{\exp(-\beta s)\cos(\omega(t - s))}{u\sqrt{s}}\exp\left(-\frac{y^2}{4\alpha u} - u\right) I_1\left(2\sqrt{\beta u s}\right) ds\,du$$
$$= -\frac{2\sqrt{\alpha\pi}}{y\sqrt{\beta}}\exp\left(-\frac{y}{\sqrt{\alpha}}\right)\cos(\omega t) + \frac{2\alpha}{y}\sqrt{\frac{\pi}{\nu}}\exp(-my)\cos(\omega t - ny) \tag{A9}$$

$$\int_0^\infty \int_0^\infty \frac{\exp(-\beta s)\sin(\omega(t - s))}{u\sqrt{s}}\exp\left(-\frac{y^2}{4\alpha u} - u\right) I_1\left(2\sqrt{\beta u s}\right) ds\,du$$
$$= -\frac{2\sqrt{\alpha\pi}}{y\sqrt{\beta}}\exp\left(-\frac{y}{\sqrt{\alpha}}\right)\sin(\omega t) + \frac{2\alpha}{y}\sqrt{\frac{\pi}{\nu}}\exp(-my)\sin(\omega t - ny) \tag{A10}$$
